# Physiological and behavioural responses of moose to hunting with dogs

**DOI:** 10.1093/conphys/coaa122

**Published:** 2020-12-30

**Authors:** Anne Randi Græsli, Luc Le Grand, Alexandra Thiel, Boris Fuchs, Olivier Devineau, Fredrik Stenbacka, Wiebke Neumann, Göran Ericsson, Navinder J Singh, Timothy G Laske, Larissa T Beumer, Jon M Arnemo, Alina L Evans

**Affiliations:** 1 Department of Forestry and Wildlife Management, Faculty of Applied Ecology and Agricultural Sciences, Inland Norway University of Applied Sciences, Koppang, Norway; 2 Department of Wildlife, Fish and Environmental Studies, Faculty of Forest Sciences, Swedish University of Agricultural Sciences, Umeå, Sweden; 3 Department of Surgery, University of Minnesota, Minneapolis, MN, USA; 4 Department of Bioscience, Aarhus University, Roskilde, Denmark

**Keywords:** Activity, *Alces alces*, body temperature, GPS (global positioning system), heart rate, human disturbance

## Abstract

Optimal management of hunted species requires an understanding of the impacts of hunting on both individual animal and population levels. Recent technological advancements in biologging enable us to obtain increasingly detailed information from free-ranging animals, covering longer periods of time, and providing the data needed to assess such impacts. In Sweden, more than 80 000 moose are harvested annually, mostly hunted with the use of baying dogs. The effects of this hunting method on animal welfare and stress are understudied. Here, we evaluated 6 real and 17 experimental hunting approaches with baying dogs [wearing global positioning system (GPS) collars] on 8 adult female moose equipped with ruminal temperature loggers, subcutaneous heart rate (HR) loggers and GPS collars with accelerometers. The obtained data were used to analyse the behavioural and physiological responses of moose to hunting with dogs. Successful experimental approaches (moose and dog were within 240 m for >10 min) resulted in higher maximum body temperature (T_b,_ 0.88°C higher) and a mean increase in HR of 24 bpm in moose at the day of the approach compared to the day after. The moose rested on average >90 min longer the day after the approach compared to the day of the approach. The moose travelled on average 4.2 km longer and had a 1.3 m/s higher maximum speed the day of the approach compared to the day after. Our results demonstrate that hunting with dogs increase moose energy expenditure and resting time (and consequently decrease time available for foraging) on an individual level. This could possibly affect body condition and reproduction rates if the hunting disturbances occur frequently.

## Teaser

Biologging devices (sensors collecting data) were used to investigate the physiological and behavioural responses of moose to hunting with baying dogs. Moose increased their heart rate, body temperature and movement rate at the day of the approach and compensated by increasing their resting time the day after the approach.

## Introduction

Understanding how human disturbance affects behaviour and physiology of animals is important for wildlife conservation and management (Wikelski and Cooke, 2006). Human disturbance may induce both acute and chronic stress. Stress is a nonspecific response to challenges in the body’s homeostasis, which can be either positive or negative depending on the type and length of the exposure (McLaren *et al.,* 2007, Reeder and Kramer, 2005, Rehbinder, 1990). A stressor is a stimulus causing stress, and the stressor could be physical, psychological or both (Reeder and Kramer, 2005). As a response to a stressor, the sympathetic nervous system is activated immediately, also known as the fight-or-flight response, while the hypothalamic–pituitary–adrenal axis is activated more slowly and lasts longer (Reeder and Kramer, 2005, Rehbinder, 1990). Stress results in physiological (i.e. metabolic, neuroendocrine and immunological) and behavioural responses of the animal, and objective and quantitative measurements of stress are often used to assess animal welfare (McLaren *et al.,* 2007). Both immediate and long-term responses to stressful situations could result in life-threatening consequences, including hyperthermia in acute stress situations and immune suppression, loss of body weight and decreased reproductive rates in long-term situations (Moberg, 2000).

In Sweden, more than 80 000 moose (*Alces alces*) are harvested annually. The hunting season lasts for 5 to 6 months from the beginning of September or October until January or February, depending on local regulations (Lavsund *et al.,* 2003; Länsstyrelserna, 2020). Hunting with dogs, especially baying dogs, is a common practice (Ball *et al.,* 1999; Heberlein, 2000). Concerns about animal welfare and stress related to hunting have been raised (Nelson *et al.,* 2005, Paquet and Darimont, 2010), but so far, data are lacking to assess these issues. Different physiological and behavioural variables help to measure and to determine if hunting disturbances result in increased energy expenditure.

Stress can be measured through physiological variables like body temperature (T_b_) and heart rate (HR) (McLaren *et al.,* 2007). However, the identification of changes during potentially stressful situations requires a baseline for these variables. Continuous measurements of HR and T_b_ could indirectly assess the sympathetic nervous system and the acute stress response as the activation results in the release of catecholamines (adrenaline and noradrenaline), which increases HR and T_b_. Increased HR (>200 bpm) and T_b_ (>42.0°C) have been recorded in brown bears (*Ursus arctos*) chased by baying dogs and black bears (*Ursus americanus*) hunted using bait (Laske *et al.,* 2011; Støen *et al.,* 2018). Extremely high T_b_ is cytotoxic, and even short periods of exposure to high T_b_ can be lethal (Lepock, 2003, Tansey and Johnson, 2015). Increased HR during hunting situations could be due to the combination of both fleeing and stress, and it is difficult to differentiate the role of each as both are normal physiological reactions. Persistently elevated HR can be dangerous when lasting for a long time, and even fatal, due to arrhythmias, infarction or myopathy of the heart (McLaren *et al.,* 2007; Stephenson, 2007).

Behavioural responses to stress caused by hunting, also known as anti-predator behaviour, have been studied in different game species including moose (Baskin *et al.,* 2004; Neumann *et al.,* 2009; Ericsson *et al.,* 2015b; Sand *et al.,* 2016), red deer (Jarnemo and Wikenros, 2014, Sunde *et al.,* 2009), roe deer (*Capreolus capreolus*) (Benhaiem *et al.,* 2008, Cederlund and Kjellander, 1991) and brown bears (Ordiz *et al.,* 2013; Hertel *et al.,* 2016). In ungulates, displacement from typical home range, increased home range area and nocturnal behaviour and stay, fight or flee when attacked are examples of anti-predator strategies (Benhaiem *et al.,* 2008, Cederlund and Kjellander, 1991, Jarnemo and Wikenros, 2014, Lingle and Pellis, 2002, Stankowich, 2008, Sunde *et al.,* 2009). Predation risk can drive prey to be more vigilant and to shift to safer habitats, which can result in lower fitness due to poorer forage quality and less time spent on feeding (Brown *et al.,* 1999). How individuals respond varies among habitat characteristics, hunting pressure and hunting method (Jarnemo and Wikenros, 2014). Hunting disturbance can result in increased resting time to compensate for increased energy consumption or to recover from the exhaustion (Le Grand *et al.,* 2019).

While several studies have evaluated behavioural responses (Ericsson *et al.,* 2015b; Sand *et al.,* 2016), none have studied the physiological responses of moose to baying dogs. Behavioural studies found that exposure to baying dogs resulted in increased activity, i.e. increased maximum speed and flight distance, and in moose leaving the area after disturbance (Ericsson *et al.,* 2015b, Sand *et al.,* 2016). Sand *et al.* (2016) found differences in the flight patterns between females with and without calves, as well as differences in flight patterns and reactions of moose in response to different dogs. Behavioural impacts, together with physiological responses, have been assessed in simulated hunts with baying dogs in brown bears (Le Grand *et al.,* 2019). During experimental days, compared to control periods, the authors noted higher HRs and T_b_, with the bears also travelling longer distances and at higher speeds.

Here, we investigated the behavioural and physiological responses of moose to simulated hunting situations with baying dogs (hereafter approaches). We tested for changes in five variables; maximum T_b_, HR, Euclidean distance travelled, maximum speed of movement and time allocated to resting versus active behaviour. We measured moose response during three periods (reference period (14–20 August), day of the approach and day after the approach). Based on previous studies of the effect of hunting on moose and other game species, we tested the following predictions:

Effects on physiology; maximum T_b_ and mean HR will be higher during the day of the approach compared to the reference period and the day after the approach.

Effects of behaviour:

1. Total distance travelled (per day) will be longer and the total time spent resting (minutes inactive/day) will be lower on the day of the approach compared to the day after.
2. Maximum speed will be higher during the day of the approach compared to the day after.

## Material and methods

### Study area and animals

The study was conducted in north-eastern Sweden, in the county of Västerbotten in Nordmaling, Vännäs and Umeå municipalities. During the study years of 2017 and 2018, a total of 675 and 782 moose were shot in the three municipalities, respectively (SvenskaJägareforbundet, 2019). The study area is characterized by boreal forest, dominated by Scots pine (*Pinus silvestris*), Norway spruce (*Picea abies*) and birch (*Betula spp.*) in addition to agricultural land and marshland. The elevation level in the area ranges from 0 to 300 m.a.s.l., and the total land area of the three municipalities are 4076.2 km^2^ (SCB, 2019). During the management period 2016–2018, the estimated moose density was on average 8.2 moose per 1000 ha in the winter (i.e. after hunting) (CAB, 2019). We studied the responses of eight adult female moose (>1.5 years old) (not each of the eight females was studied every year) and 12 baying dogs of different breeds frequently used for moose hunting (Norwegian Elkhound: n = 4, Jämthund: n = 3, other breeds: n = 5). In 2017, all five females had calves in spring, and in 2018, six had calves and one was without calf in spring.

Moose were equipped with global positioning system (GPS) Plus collars (Vectronic Aerospace GmbH, Berlin, Germany), ruminal temperature and mortality transmitters (MIT; Vectronic Aerospace GmbH, Berlin, Germany) and subcutaneous HR loggers (Reveal XT; Medtronic Inc., Minneapolis, Minnesota, USA and DST centi HRT; Star Oddi, Gardabaer, Iceland) during immobilization in February 2017. Moose were immobilized with the drug combination of 50 mg xylazine (Xylased® 500 mg, Bioveta, Ivanovice na Hané, Czech Republic) and 4.5 mg etorphine (Captivon® 98 Etorphine HCl, 9.8 mg/ml, Wildlife Pharmaceuticals (Pty) Ltd, White River, South Africa), from a helicopter using a CO_2_ powered rifle (Dan-Inject, Børkop, Denmark). The immobilization procedure is described in detail elsewhere (Evans *et al.,* 2012; Lian *et al.,* 2014; Græsli *et al.,* 2020).

### Biologgers, programming and data collection

Collars included a GPS receiver, an ambient temperature recorder, a triaxial acceleration sensor, a mortality sensor, a very high frequency (VHF) transmitter and a global system for mobile communication modem (VectronicAerospace, 2019a). In addition, each collar was linked to external sensors, i.e. the MIT and proximity sensors UHF-ID tags (VectronicAerospace, 2019b; VectronicAerospace, 2019c). We adjusted collar-derived ambient temperatures according to the offsets described by Ericsson *et al.* (2015a) to represent a reliable index for the actual ambient temperatures. The acceleration sensor integrated in the collar measures activity as back–forward and right–left movement over two axes (X and Y) on a scale from 0–255 at 6 to 8 Hz, and stores average values over 5-min recording intervals. Overall activity was presented as the sum of the activity data on the X- and Y-axes, ranging from 0 to 510 (Gervasi *et al.,* 2006). GPS positions were recorded every 3 h during the study period, and we changed it manually for the day of the approach to 10-min positions. This schedule was kept until the end of the study period for that year. Additionally, in 2017, the schedule switched to 1-min positions (n = 2) when in contact with a proximity sensor (attached to a dog). All GPS positions with associated information (ambient temperature, the most recently stored ruminal temperature and proximity contact) were sent to the wireless remote animal movement (WRAM) database for storage (Dettki *et al.,* 2014). At recapture of the moose, the remaining data stored on board the collar were manually downloaded and sent to the WRAM database.

To record moose physiological responses, we deployed a MIT in the rumen as described earlier (Minicucci *et al.,* 2018; Græsli *et al.,* 2020). MITs record the ruminal temperature at 5-min intervals and transmit the information for storage to the collar unit (VectronicAerospace, 2019b). In addition, moose were fitted with surgically implanted subcutaneous HR loggers (Reveal XT and DST centi HRT). Reveal XTs continuously monitor the HR using an electrocardiogram (ECG), which converts the mean R-R interval (rate of a ventricular cycle) into HR, and store 2-min average values (Medtronic, 2017). DST centi HRT calculates a mean HR from a 4-s ECG strip with a 150 Hz measurement frequency and stores the HR with a quality index of the signal clarity and the R-R interval regularity (StarOddi, 2017). Prior to the surgery, an analgesic, meloxicam (Metacam®, Boehringer Ingelheim Vetmedica GmBH, Germany), was given subcutaneously at a dose of 0.5 mg/kg. The DST centi HRT was surgically implanted as earlier described (Græsli *et al.,* 2020), and the Reveal XT was surgically implanted subcutaneously at the left side of the most rostral part of the sternum, according to the same procedure. We programmed and activated the Reveal XT after implantation, prior to the anaesthetic reversal.

Data recorded by the collars, MITs and Reveal XTs were downloaded in the field during recaptures in February 2018 and in February 2019. The recaptures were carried out in the same way as the initial captures. During the recaptures in 2019, we surgically removed the Reveal XTs according to the same procedure as used for implantation.

The dogs included in the real hunts belonged to local hunting teams, while the experimental hunts were conducted by trained field personnel (experienced hunters) with trained hunting dogs. All dogs were equipped with Garmin T5 or DC50 Dog collars (Garmin Ltd, Olathe, Kansas, USA) and could be tracked directly via a hand-held GPS (Garmin Astro 320) (Garmin, 2019). In addition, the dogs of the local hunting teams were equipped with UHF proximity tags (Vectronic Aerospace GmbH, Berlin, Germany), which send a signal to the moose collar when in close proximity (VectronicAerospace, 2019c).

### Experimental protocol

The experimental hunts were performed after the national leash law (i.e. dogs should be kept on leash/under supervision from 1 March to 20 August) was lifted (21 August) and until the beginning of the annual moose hunt (first Monday in September, i.e. 4 September in 2017 and 3 September in 2018, Länsstyrelserna, 2020). Real hunting situations were part of the regular moose hunt in the area from 4–24 September 2017. None of the real hunts included in our study resulted in a moose being killed. The experiments were approved by the Animal Care Committee for Northern Sweden in Umeå (application numbers: Dnr A 3-16, Dnr A 28-17). Experienced hunters, hunting dogs and field personnel carried out the real and experimental hunts, while experienced field personnel, pilots and veterinarians carried out captures, handling and surgeries.

### Experimental hunts

**Figure 1:**
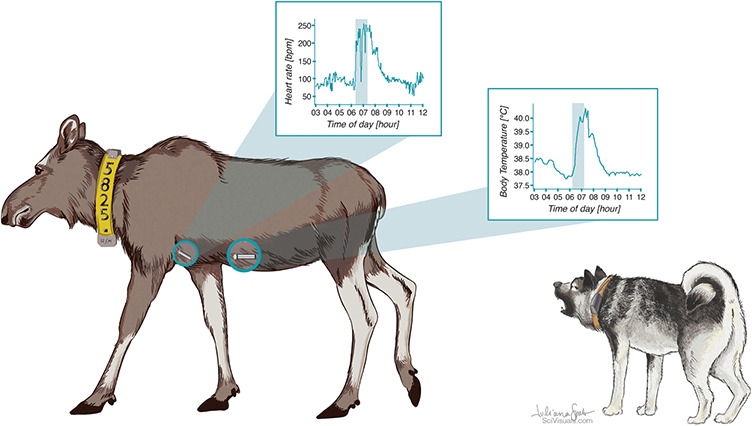
When approached by a baying hunting dog, the moose can flee in order to escape the hunting dog (as illustrated here), stand its ground or confront the dog. When the moose stands its ground the dog is typically barking and running around the moose (baying), and the hunter sneaks in and shoots the moose. Scientific Illustration by Juliana D. Spahr, SciVisuals.com (included with permission).

For each experimental hunt, a dog handler walked with the leashed dog in an upwind direction to a known moose position given by its latest GPS position. The current position of the moose was determined using a VHF receiver, aiming to release the dog close enough to the moose to avoid that the dog would pick up the scent of other, unmarked animals, while at the same time avoiding that the target moose would become aware of the human presence. Within a distance of 200 ± 50 m the dog was let off the leash and started tracking the moose while the handler monitored the dog using a hand-held GPS, thus simulating a real hunting situation (see Fig. 1). Once the dog had been in close contact with moose or was not able to find the moose, the handler leashed the dog and walked back to the car. Dog handlers participating in experimental hunts filled out a field protocol noting date and time, coordinates, moose ID, dog ID, hunting situation and weather. The dog’s GPS track was later downloaded from the hand-held GPS.

### Real hunts

When near, the UHF-ID tag of a hunting dog triggered the proximity sensor on the moose collar, and a message with the ID tag number of the dog was sent together with the GPS data from the moose. The signal strength depends on distance and topography between the UHF-ID tags and the moose collar and is expected to be between 300 and 400 meters (VectronicAerospace, 2019c). We then contacted the corresponding dog handler and met to download the tracklog of the dog, using a laptop computer and the basecamp software (Garmin Ltd, Olathe, Kansas, USA), and to collect information about the hunting situation. The field protocol was the same for both experimental and real hunts. The dog collars were programmed to collect a GPS fix every 1 s.

### Data preparation and analyses

Twenty-three dog–moose encounters (hereafter, called ‘approaches’) occurred during real (n = 6) and experimental (n = 17) hunting situations on eight individual female moose (overview in the Supplementary material (Appendix 1)). Based on the distance between the moose and the dog as well as the duration of the contact, we classified approaches as successful (moose and dog within 240 meters for at least 10 min), disturbed (moose and dog were within 240 meters for less than 10 min) and not disturbed (moose and dog were never within 240 meters). The 240-meter distance threshold was based on maximum flight initiation distance reported from a study of the same moose approached by a skier in wintertime (Viljanen, 2019), and the 10-min threshold was based on the 10-min resolution of GPS positions. We tested for differences in moose behaviour (i.e. activity) and physiology (i.e. T_b_, HR) between the day of the approach (24 h) and a 7-day reference period before the approach and to 1 day after the approach using thereby each moose as its own control. We used 1 week prior to the beginning of the dog-training period (14–20 August) as the before-approach reference period, assuming moose under non-disturbed conditions, but within similar seasonal conditions. To test for difference in moose movement, we excluded the reference period, because only GPS data at 3-h intervals were available for this period. The distance travelled per day and the speed would, therefore, have been underestimated and would have led to a bias in our model. HR, T_b_ and activity values from the reference period were generally higher than values from later in the hunting season, due to seasonal variations (Græsli *et al.,* 2020).

We only included approaches classified as successful or disturbed in the analyses and excluded approaches that had missing data (n = 4), were classified as not disturbed (n = 2), proximity sensor triggered by a dog in a car (n = 1) and approaches carried out the day before another approach on the same moose (n = 2). This resulted in data from 14 approaches (successful n = 10, disturbed n = 4) on eight individual moose. In three of these approaches, the same moose was approached twice the same day because the field personnel considered the first approach of that day as unsuccessful. Consequently, this day was considered as one approach event.

### Body temperature (T_b_)

We used a linear model from the lme4 package (Bates *et al.,* 2015) to test for difference in the maximum daily T_b_ (response variable), considering the explanatory variables Period (factor with three levels (reference period, day of approach and day after approach)) and Success (factor with two levels (disturbed and successful)). Maximum T_b_ instead of mean T_b_ was selected as the response variable to minimize the influence of drinking periods on our results, as drinking cold water drastically decreases rumen-measured T_b_ (Herberg *et al.,* 2018). Not every moose was experimentally approached repeatedly, which resulted in too few data points to include the individual moose ID as a random structure. We based model selection on Akaike’s information criterion corrected for small sample size (AICc) and carried it out with the function lCtab from the bbmle package (Bolker and R Core Team, 2017). We picked the highest ranked model with the highest AICc weight within ΔAICc ≤2 and applied a *post-hoc* test with estimated marginal means from the emmeans package (Lenth *et al.,* 2019) to estimate which levels of the categorical variables were significantly different.

### Movement of the moose

We used GPS locations collected every 10 min when analysing the movement data. In the case of missing positions or delays in switching to 10-min positions (0.08% of the positions), we used the ‘na.approx’ function from the zoo package in R to linearly interpolate the longitude and latitude values (Zeileis and Grothendieck, 2005). We excluded one of the approaches because the GPS position frequency was not appropriate as the GPS failed to switch to 10-min intervals. In total, we analysed movement data from 13 approaches on eight moose.

We used the AdehabitatLT R package to calculate the Euclidean distance between consecutive GPS positions and calculated the total distance travelled per day (m) and the maximum speed (m/min) per day (Calenge, 2006). We modelled the maximum speed and the total distance travelled per day (response variables), using a gamma-distributed generalized linear model with the identity link function. We applied Period (factor with two levels; day of approach and day after approach) and Success (factor with two levels; disturbed and successful) as explanatory variables. A random structure for individual moose ID was not included (see the explanation for the body temperature model), and the model selection followed the same approach as for body temperature.

### Heart rate

The Reveal XT calculated the HR based on recognition of R peaks in the ECG, and the ECGs were deleted (because of the storage limit) while the HR data were stored in the memory of the logger (Medtronic, 2017). The algorithm used for detection for R peaks was not always correct, likely because the Reveal XT is designed for use in human medicine, and heart anatomy and physiology of humans and moose differ. Because of that, some of the heartbeats were not detected and sometimes one heartbeat was detected as two (double counting). The absolute minimum HR of a moose resting during winter was found to be 37.5 bpm (Græsli *et al.,* 2020), so all values lower than this were removed. Based on comparisons of the HR data from the Reveal XT with manually calculated HRs from ECGs obtained by another HR logger (DST centi HRT, which was deployed additionally as part of a separate study) and accelerometer data from the same moose, we found no correlation between activity level and HR accuracy and concluded that the algorithm performed similarly during both active and passive phases. Consequently, we used the obtained raw HR data and removed unrealistically low HRs. By doing this, we were able to calculate differences in HR and identify significant changes in HR over time. However, we were unable to account for potential double counting and can therefore not report specific mean or maximum HR values.

All experiments were carried out between 08.00 and 14.00 local time, and we therefore only assessed the HR measurements during this period of the day. To compare the HR data between the different periods, we built a linear mixed-effects model (nlme package) with the raw HR data and included the variable period (reference time, day of approach and day after approach) as a fixed factor (Pinheiro *et al.,* 2019). To account for the autocorrelation within the HR data, we included the autocorrelation structure corCAR1. Moose ID was included as a random factor to account for repeated measures of the same moose. Due to the small sample size, the model could not include the explanatory variable Success and was therefore only built using the data of successful approaches.

### Resting time (active versus inactive behaviour)

To classify moose behaviour into (1) inactive (i.e. resting) and (2) active, we fitted separate hidden Markov models (HMMs) to each individuals’ observed activity data (i.e. summed acceleration of X- and Y-axes). HMMs are time-series models that assume animals’ observed movement or activity patterns to be determined by an underlying ‘hidden’ finite state sequence, where the states can be interpreted as proxies for the unobserved behavioural modes of an animal (Patterson *et al.,* 2009; Langrock *et al.,* 2012). We modelled activity using a state-dependent gamma distribution. To account for potential effects of temporal patterns and temperature on moose behaviour, the state transition probabilities were expressed as functions of the time of day, light conditions (dark, light or twilight at time of observation), day of the year and collar-derived ambient temperature using a logit link function (i.e. with the categories representing the two different states the process might switch to). We used forward selection based on Aikaike’s Information Criterion (AIC) to determine the influence of these covariates considered in each of the individual HMMs. An overview of the individuals and models chosen are presented in the Supplementary material (Appendix 2). For cyclic covariates (day of year and time of day), we included sine and cosine terms. All HMMs were fitted via numerical likelihood maximization using the momentuHMM R package (McClintock and Michelot, 2018). Each model was run with 30 sets of random starting values to avoid local maxima (always choosing the model with the highest log-likelihood value). We then determined the most likely state sequence from each HMM using the Viterbi algorithm and calculated the total time spent resting per day (min) based on these results. Subsequently, we ran a linear model with resting time as the response variable and performed the model selection, following the same approach as for body temperature.

### Behavioural responses

In order to detect flight initiation distances (FID—i.e. how close the dog was when the moose started to react behaviourally (based on collar activity (accelerometer) data) or physiologically (based on body temperature) and to estimate for how long collar activity levels and T_b_ were affected by the approaches (i.e. how long it takes for both metrics to return back to the ‘normal’ pre-approach state), we applied change point analysis (R package changepoint), (Killick and Eckley, 2014). We applied the analysis over the course of the day of the approach and used collar activity data because it was recorded at a higher resolution than the GPS data (5- vs 10-min resolution). We identified two changepoints based on the variance in the data, which should ideally correspond to the start (time of FID) and end (settling down of the moose) of the approach.

We then calculated the time the moose was affected (hereafter: time affected = time between the two changepoints), compared the times of the changepoints with the GPS data of the dogs and calculated FID (Euclidean distance). In addition, we calculated the time affected after an approach ended (the difference between the time of the second changepoint and the time the approach ended (found in the protocol)). We included approaches carried out the day before another approach on the same moose (n = 2) in the changepoint analysis. We excluded those approaches where the moose was moving before the dog found the moose (n = 2) or where data were missing (n = 1). In total, we performed changepoint analysis on 16 approaches (disturbed n = 6, successful n = 10).

All the data were prepared and analysed using R version 3.6.1 (R Core Team, 2019). We considered *P* values <0.05 as significant.

## Results

### Body temperature (T_b_), movement of the moose, heart rate and resting time

**Figure 2:**
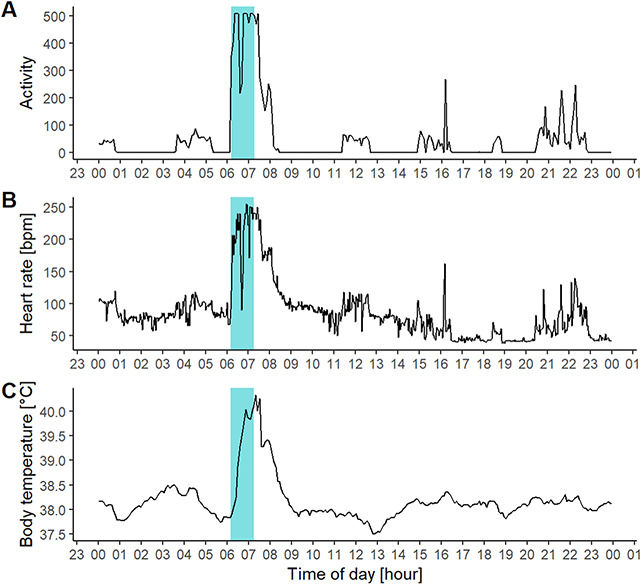
Graphical representation of (A) activity, (B) heart rate and (C) body temperature of a female moose when approached by a hunting dog during an experimental hunting approach. The blue ribbon represents the approach duration i.e. the time from the start to the end of the approach. For interpretation of the absolute heart rate values please see explanation in the main text.

**Figure 3:**
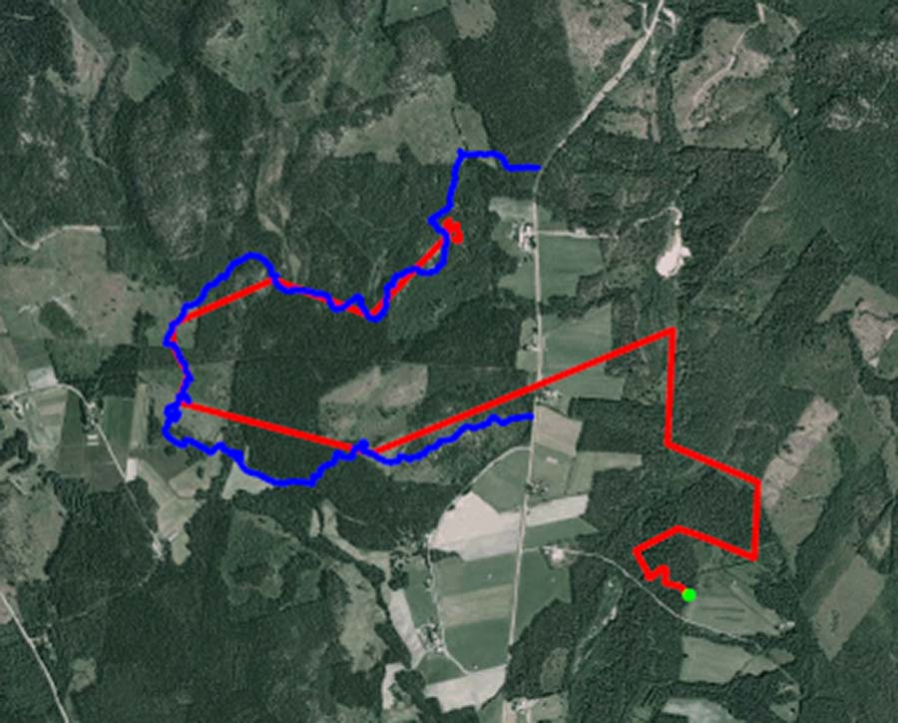
Map presenting the movement of a female moose (red line–green dot represents the last position of the moose) when approached by a hunting dog (blue line) during an experimental hunting approach.

All successfully approached moose fled away from the dogs and increased their HR, T_b_ and movement when approached (see Fig. 2). None of the moose stood its ground or confronted the hunting dogs. Hunting dogs followed moose tracks closely (e.g. Fig. 3). The highest ranked model for each response variable included period for the movement models and the interaction between period and success for T_b_ and resting (see Table 1).

**Table 1:** Log-likelihood (logLik and ΔlogLik), Akaike’s information criterion corrected for small sample size (AICc and ΔAICc), number of parameters (n) and model weight (weight) for the top-ranked, linear model combinations evaluating body temperature, movement (distance travelled and maximum speed) and resting behaviour (active vs inactive) of moose approached by hunting dogs

**Linear model combinations**	**logLik**	**AICc**	**ΔlogLik**	**ΔAICc**	**n**	**Weight**
Body temperature						
Period ^*^ success	−12.1	41.5	14.9	0.0	8	0.55
Period	−16.8	42.6	10.3	1.1	5	0.32
Period + success	−16.3	44.4	10.7	2.8	6	0.13
Distance						
Period	−235.0	477.0	7.9	0.0	4	0.49
Period ^*^ success	−232.2	477.5	10.6	0.5	6	0.38
Period + success	−234.9	479.7	7.9	2.7	5	0.13
Speed						
Period	−119.8	246.7	12.7	0.0	4	0.62
Period ^*^ success	−118.0	248.9	14.5	2.2	6	0.20
Period + success	−119.7	249.2	12.8	2.5	5	0.18
Resting						
Period ^*^ success	−201.4	420.5	11.4	0.0	8	0.56
Period + success	−205.2	422.2	7.7	1.7	6	0.24
Period	−206.7	422.6	6.1	2.2	5	0.19

**Figure 4:**
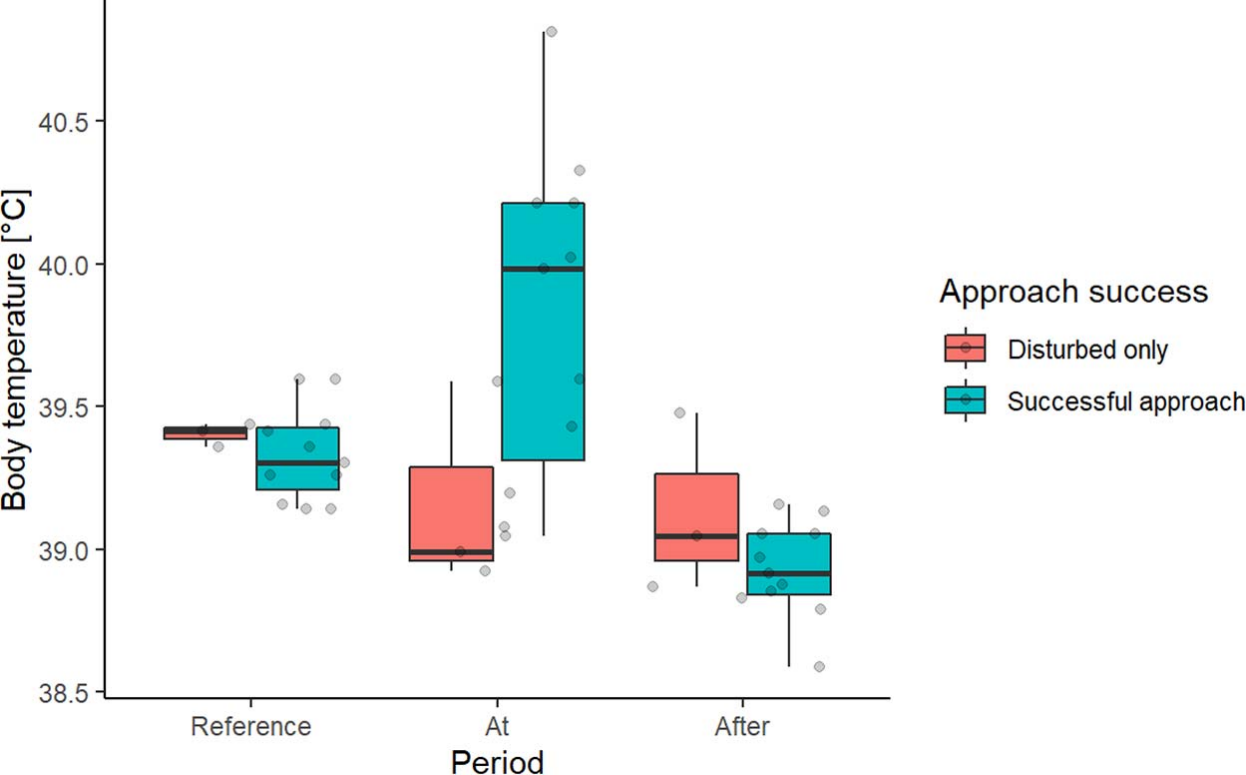
Maximum body temperature Tb (°C) during different periods (Reference; reference period (14–20 August), At; the day of the approach (24 h), After; the day after the approach (24 h)) between different success states (Disturbed only: moose and dog within 240 m for <10 min; Successful approach: moose and dog within 240 m for >10 min), for moose approached by hunting dogs.

We observed a significantly higher maximum T_b_ (0.88°C higher—SE 0.15°C, *P* value <0.001), at the day of the approach compared to the day after for the successful approaches (see Table 2 and Fig. 4). In addition, the maximum T_b_ on the day of the approach was 0.47°C higher (SE 0.15°C, *P* value 0.031) compared to the reference time. Highest T_b_ measured in the study was 40.8°C,, which was recorded during an approach. We did not detect any differences in T_b_ between the reference period, the day of the approach and the day after the approach among the moose that had been in contact with the dog <10 min (approaches classified as disturbed).

**Table 2:** Model parameter estimates, standard errors (SE), t values and *P* values for variables in the linear models evaluating body temperature, movement (distance travelled and maximum speed) and resting behaviour (active versus inactive) of moose approached by hunting dogs

	Estimate (β)	SE	*t* value	Pr(>|t|)
Body temperature				
(Intercept)	39.40	0.20	195.68	<0.001
Period at	−0.24	0.28	−0.83	0.41
Period after	−0.27	0.28	−0.96	0.34
Success successful	−0.07	0.23	−0.31	0.76
Period at: success successful	0.71	0.32	2.22	0.03
Period after: success successful	−0.13	0.32	−0.41	0.69
Distance				
(Intercept)	7123	1008	7.06	<0.001
Period after	−4145	1093	−3.79	<0.001
Speed				
(Intercept)	99.6	14.1	7.07	<0.001
Period after	−76.5	14.5	−5.28	<0.001
Resting				
(Intercept)	801.9	26.6	30.18	<0.001
Period at	−33.6	37.6	−0.89	0.378
Period after	−46.9	37.6	−1.25	0.221
Success successful	11.4	30.3	0.38	0.709
Period at: success successful	−23.2	42.8	−0.54	0.591
Period after: success successful	84.6	42.8	1.97	0.057

**Figure 5:**
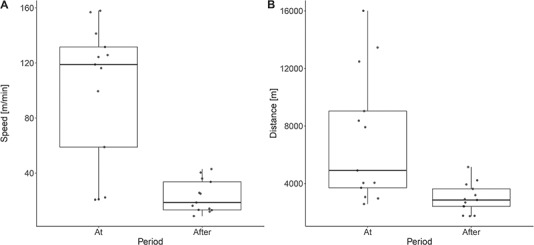
Maximum speed (m^*^min-1) (A) and overall distance travelled (m/day) (B) during different periods (At; the day of the approach (24 h), After; the day after the approach (24 h)) for moose approached by hunting dogs.

We found a higher maximum speed (mean ± SE: 76.5 ± 14.5 m/min faster; *P* value <0.001) and a longer distance travelled (mean ± SE: 4.1 ± 1.1 km longer; *P* value <0.001) at the day of the approach compared to the day after the approach for all moose (see Table 2 and Fig. 5).

**Figure 6:**
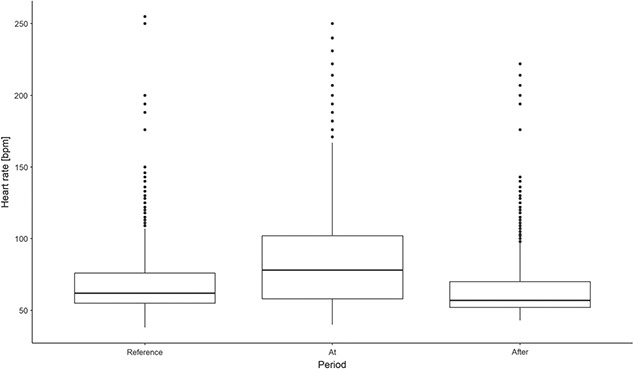
Heart rate (bpm) during different periods (Reference; reference period (14–20 August), At; the day of the approach (24 h), After; the day after the approach (24 h)), for successful approaches (moose and dog within 240 m for >10 min) of moose approached by hunting dogs. For interpretation of the absolute heart rate values please see explanation in the main text.

HR values for the day of the approach were significantly higher compared to the reference period and the day after the approach (22 bpm (SE 2 bpm, *P* value <0.001) and 24 bpm (SE 3 bpm, *P* value <0.001), respectively) (see Table 3 and Fig. 6). The highest HR measured, which could be confirmed from stored ECG episodes, was 195 bpm.

**Figure 7:**
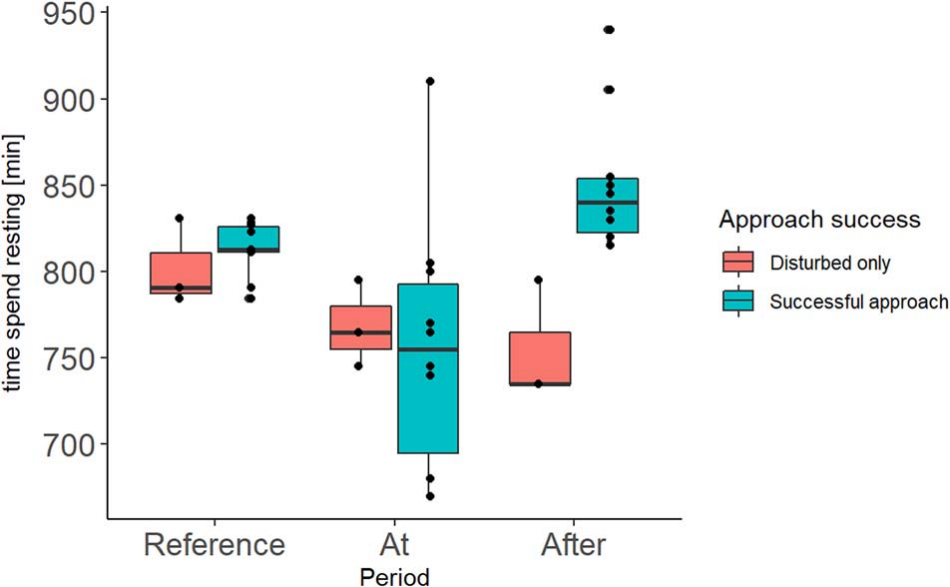
Time spent resting (minutes/day) during different periods (Reference; reference period (14–20 August), At; the day of the approach (24 h), After; the day after the approach (24 h)) and between different success states (Disturbed only: moose and dog within 240 m for <10 min; Successful approach: moose and dog within 240 m for >10 min), for moose approached by hunting dogs.

Moose rested significantly longer (94.5 min (SE 20.6 min, *P* value <0.001)) the day after a successful approach compared to the day of the approach. In addition, we demonstrated that the successfully approached moose rested 96 min longer (SE 30.3 min, *P* value 0.036) the day after the approach than the moose in approaches characterized as disturbed (moose in contact with the dog <10 min) (see Table 2 and Fig. 7). The mean resting time per day in the reference period was 13.5 h.

### Behavioural responses

Based on collar activity (accelerometer) data, we detected changepoints in 10 of the 16 approaches (disturbed n = 1, successful n = 9). Changepoints on T_b_ data were detected in three successful approaches.

For the successful approaches, the average flight initiation distance was 142 m (median) (range 86–248 m). The collar activity reached a similar level as before the approach 54 min (median) after an approach ended (range 27–114 min).

Moose T_b_ was affected 14.5 min longer than the moose activity (median total time T_b_ 132 min; range 124–160 min, versus collar activity 117.5 min; range 25–180 min). Similarly, we found that moose T_b_ was on average affected 8 min longer than activity, after an approach ended (median time T_b_ 62 min (range 36–81 min) vs activity 54 min (range 27–114 min)). Drinking episodes had an impact on the changepoint detection in T_b_ (based on graphical visualization of the T_b_ dropping around 2°C in a short period of time) after the end of an approach.

## Discussion

### Physiological and behavioural responses

This study, combining different types of biologgers, provides novel insight in the correlation between physiological and behavioural responses of moose to hunting with dogs. Combining T_b_, HR and movement data from biologgers uncovers the underlying mechanisms behind stress responses in moose and adds to the field of conservation medicine. We documented significant physiological and behavioural responses in approaches where the moose and dog had been in contact for >10 min. In contrast, we found minimal changes in behavioural and physiological parameters in approaches where the moose and dog had been in contact for <10 min (approaches classified as disturbed). Our results thus indicate that significant changes in moose behaviour and physiology dependent on the time the dog have been in close contact with the moose. However, we are aware of the small sample size of our study and that this limits the interpretation of our results.

**Table 3:** Model parameter estimates, standard errors (SE), degrees of freedom (DF), *t* values and *P* values for variables in the linear mixed model evaluating heart rate of moose approached by hunting dogs

	Value	SE	DF	*t* value	*P* value
(Intercept)	65.25	4.32	9712	15.10	<0.001
Period at	24.46	3.29	9712	7.43	<0.001
Period reference	2.85	2.49	9712	1.15	0.25

We demonstrated T_b_ in moose exceeding 40°C in the majority of the successful hunts, and the highest T_b_ measured was 40.8°C. Brown bears hunted with baying dogs are documented to have an increased T_b_ of 4.7°C from their baseline (mean) levels to the maximum T_b_ during hunting (37.5°C versus 42.2°C) (Evans *et al.,* 2016; Støen *et al.,* 2018). In moose, we found an increase of 2.3°C from the mean T_b_ baseline levels (Græsli *et al.,* 2020) to the maximum T_b_ (38.5°C versus 40.8°C). Long-time exposure to high T_b_ could be life-threatening because of cytotoxicity. Damage of mammalian cells due to hyperthermia starts after a relatively short period with temperatures >40–41°C, and the degree of damage depends on the exposure time and other stress factors (Lepock, 2003). It results in protein denaturation and impairment of the DNA synthesis in the cell, and long-term exposure leads to organ failure and death (Tansey and Johnson, 2015). Occasionally, we found a rapid decrease in rumen temperature shortly after the end of the approaches (similar to the pattern visualized by Herberg *et al.* (2018)), suggesting that some of the moose drank water when reaching the highest T_b_ levels. Drinking can, therefore, act as a behavioural thermoregulation strategy to avoid overheating (Tansey and Johnson, 2015).

We found a significant increase in HR, indicating increased energy consumption during the day of the approach. A significant correlation between HR and metabolic rate is demonstrated in a variety of animals, including moose (Green, 2011, Renecker and Hudson, 1985). The lack of validated HR measurements (not possible to validate the exact HR values) and the non-linear relationship between HR and metabolic rate in moose (Renecker and Hudson, 1985) prevented us from making a valid estimate on the increased energy consumption in this study. Even though it was not quantified, our findings (22–24 bpm difference) suggest a considerably increased energy expenditure due to hunting related disturbances when comparing our findings with seasonal differences found in moose (Græsli *et al.,* 2020). More specifically, the difference of 31.4 bpm in daily mean HR from the highest levels in summer (71.9 bpm) to the lowest daily mean in winter (40.5 bpm) represents a 60% decrease in metabolic rate from summer to winter (Græsli *et al.,* 2020). Moose might compensate for the increased energy consumption from hunting by increasing the resting time the day after, as we demonstrated. Another reason for the increased resting time is likely to be recovery from exhaustion as suggested in bears (Le Grand *et al.,* 2019). We recommend further studies to determine the effect of these events on the moose’s time spent foraging the day after a hunting situation.

Moose populations have been declining along the edges of the moose range, and increasing ambient temperatures and climate change are suggested as a reason for that (Lenarz *et al.,* 2009; Ruprecht *et al.,* 2016; Allen *et al.,* 2017). Moose are easily heat stressed with increasing ambient temperatures, resulting in increased respiratory rate, HR and body temperature (McCann *et al.,* 2013, Renecker and Hudson, 1986, Thompson *et al.,* 2019). When evaluating heat stress in moose one should consider core body temperature and the daily variations, body condition, solar radiation, vapour pressure and season in addition to ambient temperature (Thompson *et al.,* 2019; Græsli *et al.,* 2020). High ambient temperatures during the hunting season might, therefore, result in extra energy consumption in hunted moose. Increasing ambient temperatures from climate change can further result in changes in food resources and increased infection risk in addition to increased energy consumption, and all of these can result in lower survival and reproductive rates (McCann *et al.,* 2013, van Beest and Milner, 2013, van Beest *et al.,* 2012). We therefore suggest that the negative effects of hunting disturbance might be more dramatic in moose in the edges of the moose range, because of the negative effects of climate change, especially on warm hunting days.

None of the moose stayed and confronted the attacker (dog or human)—which is in line with earlier findings (Ericsson *et al.,* 2015b). In North American moose, confronting the attacker is a common anti-predator strategy towards fight off attacks from wolves (Ballard and van Ballenberghe, 2007). In Sweden, the main source of mortality for adult moose is hunting (Ericsson and Wallin, 2001). Ericsson *et al.* (2015b) suggested that female moose may have altered their anti-predator behaviour towards hunting. According to Swedish hunting laws any accompanying calf has to be shot before the female (SOU, 2009), providing a learning experience for long-living female moose. As a result, Swedish female moose might be more prone to flee compared to their North American conspecifics. Our results support previous research that hunting disturbances increase travel distance and higher maximum speed on the day of the approach compared to the day after disturbance in moose (Ericsson *et al.,* 2015b; Sand *et al.,* 2016), red deer (Jarnemo and Wikenros, 2014, Sunde *et al.,* 2009) and brown bear (Le Grand *et al.,* 2019). In red deer, it is documented that escape strategies are linked to habitat type: red deer in fragmented and more open landscapes fled more often for longer distances, and at a higher speed, than red deer in homogenous forest landscape (Jarnemo and Wikenros, 2014, Sunde *et al.,* 2009). It is likely that moose have the same type of response, yet, this aspect was beyond the scope of our study, and we suggest further research on the interplay between habitat type and physiological responses.

Moving in a sinuous pattern is possibly an antipredator strategy by moose, increasing the chance that the dog loses the track or switches to the track of another moose (Baskin *et al.,* 2004, Cederlund and Kjellander, 1991, Ericsson *et al.,* 2015b). Even if not quantified by our GPS analysis, the tracks of the moose and the hunting dog indicated that moose often moved in a sinuous pattern when approached. Our results might, therefore, have been more precise if we used one instead of 10-min GPS intervals when calculating the FID. Including wind as a parameter is valuable in explaining the antipredator strategy of moose and other prey species, as a variable to sense the location of the predator. Sometimes the hunting dogs picked up non-target moose instead; in these cases, the target moose was within 100 meters but did not move away even though a hunting dog was in close vicinity. These results are in contrast to the results of a study on the effects of hunting with dogs on roe deer as a non-target species, demonstrating that roe deer behaviour was significantly affected (Grignolio *et al.,* 2011). The differences between the studies might be due to different hunting strategies such as the use of different types and numbers of dogs, former experiences with hunting, species-specific behaviour and anti-predator strategies, and that some of the roe deer actually were pursued by the dogs (even if non-targeted) (Grignolio *et al.,* 2011).

About 45% of the moose observed by hunters in our study area were shot, meaning that >50% survived a hunting approach (Länsstyrelserna, 2019). There is not any data available on the number of surviving moose that have been chased within a given area. Yes, we assume that the amount of stress from the hunting approaches, for a given surviving moose, will vary and depends on the length of the chase, if moose were aware of hunters/dogs, their previous hunting experience, and if any in their company were shot. In spite of the documented overlap in oestrus and moose hunt in southern Sweden (Malmsten *et al.,* 2014), moose in our study area had a fairly high and stable (same level the past 20 years) reproductive rate in 2017 (>0.7) (Ericsson and Wallin, 1999, SvenskaJägareforbundet, 2019). The slaughter weights of the moose in our study area are also stable over time (SvenskaJägareforbundet, 2019), suggesting that the hunting disturbances must be stable. Most harvest occurs in the beginning of the hunting season (Singh *et al.,* 2014). Yet, to improve moose management reducing moose density in areas with high browsing damages in winter, hunting at the end of the hunting period (in winter) is increasingly discussed. Further studies should include evaluation of the effects of hunting during wintertime when the moose display hypometabolism and compare the physiological and behavioural responses during autumn and winter.

## Conclusion

Based on the physiological and behavioural results, we can conclude that hunting with baying dogs represents a notable stress event for the individual moose. Moose might compensate for the increased energy consumption by increasing the resting time on the day after being chased by a hunting dog. Yet, increased frequency of hunting disturbances and higher ambient temperature due to climate change may likely increase the energy consumption and thereby enhance the risk of negative effects on reproduction and body condition. As hunting methods and breeds of hunting dogs used are continuously evolving, managers need to continue to consider the physiological consequences for moose.

## Author contributions

J.M.A., A.L.E., G.E., W.N., N.J.S., B.F., F.S. and A.R.G. designed and initiated the study. Data collection was performed by A.R.G., A.L.E., F.S., J.M.A. and B.F. T.G.L.contributed with input for data collection and provided equipment. Data management was done by A.T., B.F., L.G. and A.R.G. A.T., L.G. and A.R.G. did the statistical analysis with advice from B.F., O.D. and L.T.B. A.R.G. led the writing of the manuscript. All authors reviewed the manuscript and approved the submitted version.

## Funding

This work was supported by Miljødirektoratet (the Norwegian Environmental Agency, grant numbers 10040125 and 16040078). Inland Norway University of Applied Sciences provided salary for A.R.G. The study was in collaboration with the research program ‘Beyond Moose—ecology and management of multispecies ungulate systems’. Beyond Moose is financially supported by the Swedish Environmental Protection Agency, Kempestiftelserna, the Swedish Association for Hunting and Wildlife Management (Västerbotten county’s älgvårdsfonden) and SLU’s Faculty of Forest Sciences.

## Conflict of interest

T.G.L. is an employee of Medtronic Inc. The remaining authors declare that the research was conducted in the absence of any commercial or financial relationships that could be construed as a potential conflict of interest.
